# Providing care to relatives with mental illness: reactions and distress among primary informal caregivers

**DOI:** 10.1186/s12888-016-0786-9

**Published:** 2016-03-25

**Authors:** Sherilyn Chang, Yunjue Zhang, Anitha Jeyagurunathan, Ying Wen Lau, Vathsala Sagayadevan, Siow Ann Chong, Mythily Subramaniam

**Affiliations:** Research Division, Institute of Mental Health, 10 Buangkok View, Singapore, 539747 Singapore

**Keywords:** Caregiver, Caregiver Reaction Assessment, Caregiving reaction, Distress, Mental health, Burden

## Abstract

**Background:**

The responsibility of caring for relatives with mental illness often falls on the family members. It has been reported that the reactions to or consequences of providing care are what rendered the role of a caregiver challenging and hence a source of distress. This present study thus aimed to identify socio-demographic correlates of caregiving experiences using the Caregiver Reaction Assessment (CRA) and to examine the associations between reactions to caregiving and psychological distress.

**Methods:**

A total of 350 caregivers with relatives seeking outpatient care at a tertiary psychiatric hospital were recruited for this study. Distress among caregivers was assessed using the Patient Health Questionnaire (PHQ-9). The CRA was administered to measure reactions from caregiving in four domains including impact on schedule and health (ISH), impact on finance (IF), lack of family support (LFS) and caregiver esteem (CE). Participants also completed a questionnaire that asked for their socio-demographic information. Multivariable linear regression analysis was first used with domains of CRA as outcome variables and socio-demographic variables as predictors in the models. The next set of multivariable linear regression analysis tested for the association between CRA domains and distress with CRA domain scores as outcome variables and PHQ-9 score as predictor, controlling for socio-demographic variables.

**Results:**

Socio-demographic correlates of CRA domains identified were age, education, employment, income and ethnicity. Domain scores of CRA were significantly associated with PHQ-9 score even after controlling for socio-demographic variables. A higher distress score was associated with greater impact felt in the domain of ISH (β = 0.080, *P* < 0.001), IF (β = 0.064, *P* < 0.001), and LFS (β = 0.057, *P* < 0.001), and was associated with lower CE domain scores (β = −0.021, *P* < 0.05).

**Conclusion:**

This study identified several socio-demographic correlates of caregiving reaction in the different domains. Each of these domains was found to be significantly associated with caregiver distress. Higher distress was associated with stronger impact on the negative domains and a lower impact in the positive domain of caregiving reaction. Interventions such as educational programs at the caregiver level, and also promoting wider social care support in these domains may help to address caregiver distress.

## Background

Mental illness can be disabling and limits patients’ functionality in different domains of life. Care is thus often required for these patients. With the advent of deinstitutionalization as recommended by the World Health Organization [1], the responsibility of care has then shifted mainly to the informal caregivers of the patients [2].

Informal caregivers suffer from psychological distress due to their caregiving roles and elevated depressive symptoms are often a manifestation of distress [3]. The impact of caregiving on both the physical and mental health of caregivers is well documented. Compared to non-caregivers, caregivers were more depressed, had higher levels of stress, more outpatient visits and a poorer quality of life [4–7]. Depressive symptoms among distressed caregivers were independently associated with physical morbidity and mortality [8–10]. There is also some evidence to support a causal relationship between caregiving and subsequent onset of clinical depression and anxiety disorders [11]. Furthermore, the negative effects were not only felt by caregivers; there were ramifications for care recipients as well. Longitudinal studies found that caregiver distress was predictive of early discontinuation of care for disabled elderly [12] and institutionalization of patients with dementia [13, 14]. Studies have also reported depressive symptomology in caregivers as a factor that could compromise the quality of care given [15–17]. Notwithstanding the detrimental effects of caregiving, there are positive outcomes from having to care for one’s relatives- gratitude and appreciation from patients, improved family cohesion, developing resilience, and gaining a sense of self-worth and accomplishment [18–20]. These positive aspects of caregiving, in turn, have been associated with lower levels of caregiver burden and depression [21].

A study by Jarvis et al. found that it was the reactions to and the consequences of caregiving, rather than performing the actual task, that posed challenges for the caregivers [22]. For example, caregivers might not have issues with the act of accompanying their relative for medical consultation, but the cost of having to change their schedule and face disruption in work and daily routine can make the caregiving task challenging. The lack of family assistance during such instances might further aggravate the situation and hence these reactions can impair caregivers’ ability and willingness to provide care for their relatives.

The Caregiver Reaction Assessment (CRA) developed by Given et al. provides a multidimensional measure of caregiver reaction and it captures both positive and negative aspects of caregiving [23]. The instrument has been validated among caregivers of people with chronic physical and mental illness [24–26]. Various socio-demographic correlates of caregiving experiences had been identified, including age, gender, relationship with patient, marital status, income and education level of caregivers [27–30]. The instrument was also used to assess the association between reaction to caregiving and mental health of caregivers. In a study across eight European countries, significant correlations were found between domains of the CRA and psychological well-being and quality of life [31]. Caregivers of geriatric care recipients who reported better mental health had fewer negative reactions and more positive reactions from caregiving [27]. A study examining depression among informal caregivers of elderly reported that negative reactions from caregiving were collectively found to be the key factor in predicting caregiver depressive symptoms [32]. In another study, Aggar et al. found all domains of the CRA to be significantly correlated with depression and anxiety scores. The subsequent regression analysis found that the domains on the impact on caregivers’ schedule and health were significant predictors of caregiver depression [33]. However, socio-demographic factors which might have influenced this association were not accounted for in the analysis.

In Singapore, a multi-ethnic country located in Southeast Asia, the notion of filial piety is often manifested in the form of providing care to family members who are unwell or have aged [34]. Knowing that the responsibility of care is shouldered mostly by these informal caregivers, it is therefore important to study how caregivers can be affected by their caregiving experiences. Results from this study can provide valuable insights for healthcare professionals and also policymakers on specific challenges that caregivers in Singapore are facing and to allocate appropriate support services. This study thus aimed to examine socio-demographic correlates of caregiving reactions and the associations of these experiences with caregiver psychological distress.

## Methods

### Study design, setting and participants

Participants of this cross-sectional epidemiology study were primary caregivers of outpatients seeking treatment at the Institute of Mental Health (IMH), which is a tertiary psychiatric hospital in Singapore, and its satellite clinics. For the purpose of discussion, caregiving in this article refers to primary informal caregiving that is unpaid care for patients by their family members rather than by a paid professional caregiver. A total of 350 primary caregivers of people with schizophrenia and related psychosis, major depressive disorder, anxiety disorders (generalized anxiety disorder and obsessive-compulsive disorder) or dementia were recruited through convenience sampling. The primary caregiver was defined as the person whom the patient depended on the most and who had been staying with and caring for the patient for at least 6 months. To be included in the study, the caregiver had to be a Singapore Citizen or Permanent Resident, aged 21 years and above, and able to read and comprehend English, Chinese, Malay or Tamil. Participants who spoke only dialects were excluded from the study. Participants completed the questionnaire in the language they felt most comfortable with and were reimbursed for their participation upon completion of the questionnaire. The study was approved by the National Healthcare Group Domain Specific Review Board in Singapore and written informed consent was obtained from the participants.

### Measures

Reactions to caregiving were operationalized using the CRA and the original scale consisted of 24 items that assessed five domains of reactions to caregiving- negative impact on health, disrupted schedule, financial problems, lack of family support and a positive domain of caregiver esteem [23]. Participants scored the items on a 5-point Likert scale (1 = “*strongly disagree*” to 5 = “*strongly agree*”) and a total score was obtained on each subscale by taking the means of the items; a higher score indicates a stronger impact in that domain. Multiple studies have validated the CRA and a number have reported a high overlap between the domain on impact on health and schedule [24–26, 35–38]. In a validation study conducted in Singapore among caregivers of elderly with functional limitation, Malhotra et al. found a 4-factor structure which combined the domains of health and schedule. The resulting four factors were: impact on schedule and health (ISH); impact on finance (IF); lack of family support (LFS); and caregiver esteem (CE) [38]. Our analysis showed a similar factor structure, though with slight differences in item loadings and in the total number of items. The analysis in this study used the 4-factor structure and item loadings that we had found. Reliability of the CRA in this study sample was tested using Cronbach’s alpha and the alpha values for the domain on ISH (α = 0.81), IF (α = 0.71), LFS (α = 0.79) and CE (α = 0.83) were obtained. Alpha values greater than 0.7 were generally considered as acceptable and above 0.8 as good [39].

Psychological distress of caregivers was assessed using the Patient Health Questionnaire (PHQ-9) [40]. The PHQ-9 is a 9-item instrument that has been widely used for screening and assessing symptom severity of depression and it is based on the Diagnostic and Statistical Manual of Mental Disorder IV (DSM-IV) [41] diagnostic criteria. Participants scored the frequency of symptoms on a scale from “0” (*not at all*) to “3” (*nearly every day*), giving a total depression severity score ranging from 0 to 27 with higher scores indicating a greater symptom severity. PHQ-9 score of ≥10 has been commonly used as a cut-off point that warrants for professional support and further evaluation of depressive disorder. A systematic review of studies using PHQ-9 had concluded on its sound psychometric properties and diagnostic validity [42]. The instrument displayed high internal reliability in this study sample with a Cronbach’s alpha of 0.88.

Socio-demographic information of the caregivers was also collected, including information on age, gender, ethnicity, marital status, highest level of education, employment status, income level (measured in Singapore Dollars), relationship with care recipient and presence of medical condition.

### Statistical analysis

Analysis in this study was performed using Statistical Package for Social Sciences (SPSS) version 23. Descriptive statistics were used to describe frequency distribution of study sample and CRA domain scores. To examine the socio-demographic correlates of reactions to caregiving, multiple linear regressions were used with each CRA domain score as outcome variables and socio-demographic variables as predictors. Next, normality of scores was checked for and Spearman’s rank correlation was used to test the associations between CRA domains and PHQ-9 score. Multivariable linear regression was then conducted to examine the association between CRA domains and PHQ-9 score. Socio-demographic variables including age, gender, ethnicity, highest level of education attained, employment status, marital status, income level, presence of medical condition, and relationship with care recipient were included in the regression models to account for confounding effects. A total of four models were tested where each CRA domain was treated as the outcome variable, and PHQ-9 score as covariate in the model controlling for socio-demographic factors. Collinearity among the variables was checked prior to running the regression analyses. All statistically significant results were reported at *P* ≤0.05.

## Results

### Sample characteristics and distribution of CRA scores

Six participants were withdrawn from the study due to their failure to complete the questionnaire, resulting in a total of 344 cases that were analysed. Most of the caregivers were in the age group of 50–64 years (45.6 %). A majority of them were females (68.0 %), of Chinese ethnicity (57.6 %), employed (64.2 %) and were parents of the care recipient (35.2 %; Table 1). Mean scores obtained were 2.97 for the ISH domain, 2.91 for the IF domain, 2.52 for the LFS domain, and 4.00 for the CE domain. Caregivers scored the highest in the positive domain of caregiver esteem, followed by the negative domain on the impact on schedule and health.

**Table 1:** Caregiver characteristics of study sample (*n* = 344)

		*n*	%
Age group	21–34	65	18.9
	35–49	82	23.8
	50–64	157	45.6
	65 & above	40	11.6
Gender	Male	110	32.0
	Female	234	68.0
Ethnicity	Chinese	198	57.6
	Malay	66	19.2
	Indian	75	21.8
	Others	5	1.5
Education	No formal & primary	52	15.1
	Secondary	148	43.0
	Vocational	20	5.8
	Pre-U/Junior College/Diploma	68	19.8
	University & above	56	16.3
Employment status	Employed	221	64.2
	Economically inactive	94	27.3
	Unemployed	29	8.4
Marital status	Single	89	25.9
	Married	223	64.8
	Divorced/Separated/Widowed	32	9.3
Monthly income (SGD)	No income	97	28.2
	Below S$2000	115	33.4
	S$2000–S$3999	79	23.0
	S$4000 and above	47	13.7
	Missing data	6	1.7
Relationship (with ref. to care recipient)	Spouse	80	23.3
	Parent	121	35.2
	Son/Daughter	89	25.9
	Sibling	37	10.8
	Others	16	4.7
	Missing data	1	0.3
Any medical condition	Yes	134	39.0
	No	210	61.0

### Socio-demographic correlates of CRA domains

Table 2 presents the results from the multivariable regression analyses to examine socio-demographic correlates of caregiving experiences. Compared to the youngest age group, being a caregiver within the ages of 35–49 years was associated with greater impact in the domain of health and schedule (β = 0.313). Caregivers with lower education (i.e. pre-university, diploma and junior college), as compared to those with a university degree qualification and above, perceived a greater impact on finance (β = 0.390). Greater impact in the domain of finance was also associated with caregivers who were unemployed (β = 0.544). Caregivers with a monthly income of below $2000 perceived a greater lack of family support than those who were earning $4000 and above (β = 0.470). Ethnicity was associated with caregiver esteem domain scores. Compared to Chinese caregivers, caregivers of Indian (β = 0.233) and Malay (β = 0.226) ethnicity were associated with higher domain scores. Having secondary education, as compared to university and above qualifications, was associated with a greater impact on caregiver esteem (β = 0.360).

**Table 2:** Socio-demographic correlates of CRA domains

		Impact on schedule & health (ISH)		Impact on finance (IF)		Lack of family support (LFS)		Caregiver esteem (CE)	
		β	*P*-value	β	*P*-value	β	*P*-value	β	*P*-value
Age group	21–34	ref		ref		ref		ref	
	35–49	0.313	0.021^a^	0.097	0.541	0.170	0.255	−0.065	0.553
	50–64	0.108	0.448	−0.241	0.151	0.176	0.262	0.058	0.613
	65 & above	0.046	0.805	−0.192	0.385	0.151	0.465	−0.091	0.551
Gender	Male	−0.042	0.674	0.066	0.571	−0.048	0.664	0.067	0.408
	Female	ref		ref		ref			
Ethnicity	Chinese	ref		ref		ref		ref	
	Malay	−0.196	0.075	−0.024	0.854	−0.091	0.451	0.226	0.011^a^
	Indian	−0.015	0.885	−0.003	0.981	0.031	0.786	0.233	0.005^a^
	Others	0.023	0.947	−0.124	0.756	0.257	0.492	−0.323	0.241
Education	No formal & primary	0.005	0.977	0.243	0.237	−0.104	0.588	0.115	0.417
	Secondary	0.004	0.980	0.196	0.248	−0.004	0.979	0.360	0.002^a^
	Vocational	−0.081	0.699	0.450	0.070	−0.169	0.465	0.034	0.844
	Pre-U/Junior College/Diploma	0.176	0.215	0.390	0.020^a^	0.012	0.938	0.131	0.254
	University & above	ref		ref		ref		ref	
Employment status	Employed	ref		ref		ref		ref	
	Economically inactive	−0.234	0.178	0.009	0.964	−0.154	0.421	0.088	0.532
	Unemployed	0.024	0.902	0.544	0.018^a^	0.084	0.694	0.114	0.471
Marital status	Single	−0.073	0.685	−0.072	0.733	−0.139	0.481	0.036	0.805
	Married	−0.012	0.935	0.087	0.612	−0.017	0.916	−0.096	0.416
	Divorced/Separated/Widowed	ref		ref		ref		ref	
Monthly income (SGD)	No income	0.334	0.132	0.200	0.440	0.461	0.058	−0.296	0.099
	Below S$2000	0.182	0.246	0.302	0.102	0.470	0.007^a^	−0.228	0.073
	S$2000–S$3999	0.087	0.558	0.110	0.527	0.276	0.091	−0.216	0.073
	S$4000 and above	ref		ref		ref		ref	
Relationship (with ref. to care recipient)	Spouse	ref		ref		ref		ref	
	Parent	−0.055	0.657	0.243	0.093	−0.193	0.153	−0.061	0.536
	Son/Daughter	−0.031	0.830	0.270	0.108	0.140	0.372	−0.091	0.430
	Sibling	−0.097	0.591	0.254	0.229	0.212	0.284	−0.228	0.117
	Others	−0.001	0.998	−0.233	0.348	0.239	0.302	0.108	0.526
Any medical condition	Yes	0.109	0.222	0.061	0.557	0.076	0.436	−0.083	0.251
	No	ref		ref		ref		ref	

^a^Results are statistically significant at *P* <0.05

### CRA associations with caregiver distress

In the univariate analysis, all fours domains were significantly correlated with distress scores (Table 3). After accounting for socio-demographic factors, the associations between all CRA domains and the PHQ-9 remained significant in the multivariable linear regression (Table 4). A higher distress score was associated with greater impact on schedule and health (β = 0.080, *P* < 0.001), greater impact on finance (β = 0.064, *P* < 0.001), greater lack of family support (β = 0.057, *P* < 0.001), and a lower caregiver esteem (β = −0.021, *P* < 0.05).

**Table 3:** Correlations between CRA domains and PHQ-9 score

	Spearman’s rho			
	Impact on schedule & health	Impact on finance	Lack of family support	Caregiver esteem
PHQ-9 score	0.536^a^	0.405^a^	0.315^a^	−0.154^a^

^a^Results significant at the 0.01 level

**Table 4:** Associations between CRA domain scores and PHQ-9 score using multivariable linear regression

PHQ-9 score	β	S.E.	*P*-value	95 % Confidence interval	
				Lower	Upper
CRA domains					
Impact on schedule & health	0.080	0.007	<0.001^a^	0.067	0.093
Impact on finance	0.064	0.009	<0.001^a^	0.047	0.081
Lack of family support	0.057	0.008	<0.001^a^	0.041	0.074
Caregiver esteem	−0.021	0.006	0.001^a^	−0.033	−0.008

Socio-demographic variables including age, gender, ethnicity, marital status, education, employment, income, relationship with care recipient and presence of medical condition were controlled for in each of the four regression models where CRA domain was the outcome variable

^a^Results are statistically significant at *P*<0.05

## Discussion

Several socio-demographic correlates of reactions to caregiving were identified in this study including age, education level, employment status, income, and ethnicity. All four domains of caregiving reactions: impact on schedule and health, impact on finance, lack of family support, and caregiver esteem were associated with psychological distress as assessed by the PHQ-9. The associations remained significant even after controlling for socio-demographic variables. Some of the findings reported here are consistent with results from other studies in the literature [27, 28].

In our study, caregivers in the age group of 35–49 years perceived significantly higher impact in terms of disrupted schedule and affected health as compared to caregivers between 21 and 34 years old. One possible explanation could be that caregivers in this age group are more likely to have taken on multiple social roles with greater commitments. Besides being a caregiver, he/she might be a parent, a spouse and an employee with more responsibilities. Demands and responsibilities stemming from these various assumed roles might have contributed to the perceived impact of caregiving on schedule and health [43].

Being unemployed was associated with stronger impact in the domain of finance and this association had taken into account the level of income earned. Additional analysis conducted found that unemployed caregivers also perceived greater impact on finance as compared to economically inactive caregivers. Caregivers categorized as economically inactive in this study were housewives, students or retired individuals who probably felt less of an obligation to be employed and to contribute to caregiving expenses. This might suggest that rather than the financial resources, it was the expectation or the obligation of the caregiver to have a job that contributed to the impact felt.

Caregivers earning less than $2000 a month felt a greater lack of family support than caregivers who were earning $4000 and above. Given that past literature review found respite care services beneficial for caregivers [44] and another study that reported an inverse association between receiving assistance from a foreign domestic helper and negative reaction from caregiving [32], a plausible explanation to the association could be that caregivers in the former category were not as financially competent to engage such additional help for the caregiving task, and thus required more assistance from their family members. Future studies can look into the pathways through which having help from external support in the form of respite care services or domestic helpers can reduce negative reactions to caregiving.

An interesting ethnic difference emerged in the positive domain of reactions to caregiving. Compared to caregivers of Indian and Malay ethnicity, Chinese caregivers perceived lower caregiver esteem. One way of looking at this may be through the construct of filial piety (i.e. respect and care for one’s elders). Although this construct originated from traditional Chinese Confucian values, there is evidence that it is not unique to Chinese culture. A study found that Chinese Singaporeans endorsed traditional values such as filial piety less than their non-Chinese counterparts (i.e. Malays and Indians) [45]. Additionally, a study examining filial piety among Chinese in Singapore pointed out the symbolic status of the construct as a possible mere display for the purpose of ‘face saving’ in certain cases [46]. Taking these findings in context, it suggests that Chinese caregivers might have a stronger sense of obligation to provide care for their relatives and this could have influenced their feeling of esteem in that providing care was not of altruistic intention but out of duty. Having a stronger sense of obligation was in fact found to be positively associated with subjective burden in caregivers [47].

Another main finding from this study was that each of the domains of caregiving was associated with caregiver distress. It is possible that caregivers who were distressed had poorer coping abilities and hence had stronger reactions from caregiving. It was found that caregivers with higher depressed mood had more difficulties performing physical tasks [48], which might have affected their ability to participate in activities that promote their health and overall well-being. It is also plausible to conceive that caregivers with elevated distress were unable to manage their finances or to effectively communicate their needs to family members. The association can also be understood using Pearlin et al’s stress process model where caregiving duties act as secondary role strains that are in conflict with other domains of life, thus causing an impact on the mental well-being of caregivers [49]. For instance a reduction in engagement in social and physical activities due to competing time, which could have attenuating effects on chronic stress from caregiving, can possibly precipitate depressive symptoms in caregivers [50–52].

Findings from this study have several important implications for caregivers. Firstly, our results would suggest that educating caregivers on effectively managing their schedule, attending to their health issues, addressing their financial needs, facilitating support from their family members in caregiving tasks, and enhancing their esteem might be useful in reducing their distress. For example, health awareness initiatives can help to draw attention to the impact of caregiving on caregiver’s mental well-being and possible ways to alleviate the effects. Given the findings from this study, such initiatives could target caregivers with certain demographics- those in the late thirties to forties and those who are unemployed as they were caregivers who perceived greater impact from caregiving. Chinese caregivers could also potentially benefit from interventions that serve to enhance the way they appraise their caregiving role and thereby increasing their esteem as a caregiver. This could be attained through positive encouragement and acknowledgement of the contributions made by caregivers in improving the lives of patients, possibly through affirmative communication with healthcare professionals or even their own family members [53, 54]. Secondly, these efforts and initiatives should be complemented with a dissemination of information on respite care services. Given that studies have found the positive impact of respite care services on caregivers’ quality of life [55] and depressive symptomatology [56], these services can provide caregivers with a temporary relief from their caregiving duties to attend relevant activities or handle personal matters. Last but not least, psychoeducation for distressed caregivers can now be understood in the context of the impact of reactions to caregiving on their mental health. Counselling goals can be targeted to address needs in the various domains, for instance managing expectations of family members’ assistance in caregiving tasks.

Besides involving caregivers themselves as the main agent of change, the wider public and policymakers have a role to play. An increase in the public awareness of the challenges faced by caregivers can serve to generate more supportive social responses for better caregiver support, possibly advocating it as a national social agenda. For example, employers can take the lead by allowing some flexibility in the work arrangements of employees who are caregivers. This could help caregivers in managing their schedule to accommodate caregiving responsibilities. Our findings suggest that unemployed caregivers and those with lower education merit more attention due to the greater impact on these groups of caregivers. These results can thus help in crafting social policies targeted at these groups of caregivers, possibly through subsidies for paid care services or care support educational programs. Recognising that taking on a caregiver role can occur at any point in life and having policies ascribed to the role, rather than looking at the status of being a working adult, is what a care-centred policy should be like and it would be needed to provide adequate support for caregivers [57].

However, the results and implications should be considered in view of the limitations of the study. The study being a cross-sectional survey limits the ability to draw conclusions regarding causality. Patient characteristics such as age and depressive symptoms were not explored in this study which could have an impact on caregiver reactions [30, 58] and distress [59, 60]. Analysis by diagnosis of patients could not be conducted due to an insufficient sample size in some diagnostic groups. The main intention of this study was to focus on caregiver characteristics rather than other factors such as patient demographics and caregiving tasks. Future studies can thus examine the effects of patient profile and caregiving tasks on caregiver reactions and distress. Given that the caregivers of our study sample co-resided with the care recipient, this may limit the generalisability of our findings to countries where co-residency among caregivers and care recipient is not a norm. This may have an effect on the hours spent on caring and subsequently the level of distress on the caregivers [61, 62]. These limitations notwithstanding, one major strength of this study lies in utilizing an instrument (i.e. the Caregiver Reaction Assessment) that was previously validated in the local setting. Additionally, participants from different ethnic groups were included in the study and this enhanced the reliability of the findings.

## Conclusions

In conclusion, this study identified several socio-demographic differences in reactions to caregiving, and that all domains of reaction were associated with distress. Having higher distress was associated with a greater impact on schedule and health, impact on finance, lack of family support and lower caregiver esteem. Initiatives with the intention of addressing needs in these aspects may help to reduce caregiver distress. It is crucial to bear in mind that caregivers are not the sole agents of action. Support should also come from the wider community and at a policy level to support this group of individuals.

### Ethics approval and consent to participate

The study was approved by the National Healthcare Group Domain Specific Review Board in Singapore and written informed consent was obtained from the participants.

### Consent for publication

Not applicable.

### Availability of data and materials

Data supporting the findings is available upon request. Please contact the Principal Investigator of this study, Yunjue Zhang (yunjue_zhang@imh.com.sg), for data availability.

## Abbreviations

CE: caregiver esteem
CRA: Caregiver Reaction Assessment
IF: impact on finance
ISH: impact on schedule and health
LFS: lack of family support
PHQ-9: Patient Health Questionnaire-9

## References

[1] World Health Organization. The world health report 2001–mental health: new understanding, new hope. Geneva; 2001

[2] Chan BW, O’Brien AM (2011). The right of caregivers to access health information of relatives with mental illness. Int J Law Psychiatry.

[3] Chung ML, Pressler SJ, Dunbar SB, Lennie TA, Moser DK, Endowed G (2010). Predictors of depressive symptoms in caregivers of patients with heart failure. J Cardiovasc Nurs.

[4] Chan A, Malhotra C, Malhotra R, Rush AJ, Ostbye T (2013). Health impacts of caregiving for older adults with functional limitations: results from the Singapore survey on informal caregiving. J Aging Health.

[5] Wong D, Lam A, Chan S, Chan S (2012). Quality of life of caregivers with relatives suffering from mental illness in Hong Kong: roles of caregiver characteristics, caregiving burdens, and satisfaction with psychiatric services. Health Qual Life Outcomes.

[6] Pinquart M, Sorensen S (2003). Differences between caregivers and noncaregivers in psychological health and physical health: a meta-analysis. Psychol Aging.

[7] Gupta S, Isherwood G, Jones K, Van Impe K (2015). Assessing health status in informal schizophrenia caregivers compared with health status in non-caregivers and caregivers of other conditions. BMC Psychiatry.

[8] Schulz R, O’Brien AT, Bookwala J, Fleissner K (1995). Psychiatric and physical morbidity effects of dementia caregiving: prevalence, correlates, and causes. Gerontologist.

[9] Schulz R, Beach SR (1999). Caregiving as a risk factor for mortality: the Caregiver Health Effects Study. JAMA.

[10] Pinquart M, Sorensen S (2007). Correlates of physical health of informal caregivers: a meta-analysis. J Gerontol B Psychol Sci Soc Sci.

[11] Dura JR, Stukenberg KW, Kiecolt-Glaser JK (1991). Anxiety and depressive disorders in adult children caring for demented parents. Psychol Aging.

[12] Arai Y, Sugiura M, Washio M, Miura H, Kudo K (2001). Caregiver depression predicts early discontinuation of care for disabled elderly at home. Psychiatry Clin Neurosci.

[13] Buhr GT, Kuchibhatla M, Clipp EC (2006). Caregivers’ reasons for nursing home placement: clues for improving discussions with families prior to the transition. Gerontologist.

[14] Cohen CA, Gold DP, Shulman KI, Wortley JT, McDonald G, Wargon M (1993). Factors determining the decision to institutionalize dementing individuals:a prospective study. Gerontologist.

[15] MacNeil G, Kosberg JI, Durkin DW, Dooley WK, DeCoster J, Williamson GM (2010). Caregiver mental health and potentially harmful caregiving behavior: the central role of caregiver anger. Gerontologist.

[16] Beach SR, Schulz R, Williamson GM, Miller LS, Weiner MF, Lance CE (2005). Risk factors for potentially harmful informal caregiver behavior. J Am Geriatr Soc.

[17] Williamson GM, Shaffer DR (2001). Relationship quality and potentially harmful behaviors by spousal caregivers: how we were then, how we are now. The Family Relationships in Late Life Project. Psychol Aging.

[18] Bauer R, Sterzinger L, Koepke F, Spiessl H (2013). Rewards of caregiving and coping strategies of caregivers of patients with mental illness. Psychiatr Serv.

[19] Cohen HL, Youjung L (2006). Dementia caregivers: rewards in multicultural perspectives. J Hum Behav Soc Environ.

[20] Schwartz C, Gidron R (2002). Parents of mentally ill adult children living at home: rewards of caregiving. Health Soc Work.

[21] Pinquart M, Sorensen S (2003). Associations of stressors and uplifts of caregiving with caregiver burden and depressive mood: a meta-analysis. J Gerontol B Psychol Sci Soc Sci.

[22] Jarvis A, Worth A, Porter M (2006). The experience of caring for someone over 75 years of age: results from a Scottish General Practice population. J Clin Nurs.

[23] Given CW, Given B, Stommel M, Collins C, King S, Franklin S (1992). The caregiver reaction assessment (CRA) for caregivers to persons with chronic physical and mental impairments. Res Nurs Health.

[24] Nijboer C, Triemstra M, Tempelaar R, Sanderman R, van den Bos GA (1999). Measuring both negative and positive reactions to giving care to cancer patients: psychometric qualities of the Caregiver Reaction Assessment (CRA). Soc Sci Med.

[25] Persson C, Wennman-Larsen A, Sundin K, Gustavsson P (2008). Assessing informal caregivers’ experiences: a qualitative and psychometric evaluation of the Caregiver Reaction Assessment Scale. Eur J Cancer Care.

[26] Grov EK, Fossa SD, Tonnessen A, Dahl AA (2006). The caregiver reaction assessment: psychometrics, and temporal stability in primary caregivers of Norwegian cancer patients in late palliative phase. Psychooncology.

[27] De Frias CM, Tuokko H, Rosenberg T (2005). Caregiver physical and mental health predicts reactions to caregiving. Aging Ment Health.

[28] Grov EK, Eklund ML (2008). Reactions of primary caregivers of frail older people and people with cancer in the palliative phase living at home. J Adv Nurs.

[29] Gohil R, Crosby-Nwaobi R, Forbes A, Burton B, Hykin P, Sivaprasad S (2015). Caregiver burden in patients receiving Ranibizumab therapy for neovascular age related macular degeneration. PLoS One.

[30] Park C-H, Shin DW, Choi JY, Kang J, Baek YJ, Mo HN, Lee M-S, Park S-J, Park SM, Park S. Determinants of the burden and positivity of family caregivers of terminally ill cancer patients in Korea. Psychooncology. 2012;21(3):282–90. 10.1002/pon.1893 22383270

[31] Alvira MC, Risco E, Cabrera E, Farre M, Rahm Hallberg I, Bleijlevens MHC, Meyer G, Koskenniemi J, Soto ME, Zabalegui A. The association between positive-negative reactions of informal caregivers of people with dementia and health outcomes in eight European countries: a cross-sectional study. J Adv Nurs. 2015;6:1417. 10.1111/jan.12528 25250659

[32] Malhotra C, Malhotra R, Østbye T, Matchar D, Chan A (2012). Depressive symptoms among informal caregivers of older adults: insights from the Singapore Survey on Informal Caregiving. Int Psychogeriatr.

[33] Aggar C, Ronaldson S, Cameron ID (2010). Reactions to caregiving of frail, older persons predict depression C. Aggar et al. Caregiving reactions predict depression. Int J Ment Health Nurs.

[34] Kyu-Taik S (1998). An exploration of actions of filial piety. J Aging Stud.

[35] Ge C, Yang X, Fu J, Chang Y, Wei J, Zhang F, Nutifafa AE, Wang L. Reliability and validity of the Chinese version of the Caregiver Reaction Assessment. Psychiatry Clin Neurosci. 2011;65(3):254–63. 10.1111/j.1440-1819.2011.02200.x 21507132

[36] Yang H-K, Shin DW, Kim S-Y, Cho J, Chun S-H, Son KY, Park B, Park J-H. Validity and reliability of the Korean version of the Caregiver Reaction Assessment Scale in family caregivers of cancer patients. Psychooncology. 2013;22(12):2864–8. 10.1002/pon.3364 23913745

[37] Misawa T, Miyashita M, Kawa M, Abe K, Abe M, Nakayama Y, Given CW. Validity and reliability of the Japanese version of the Caregiver Reaction Assessment Scale (CRA-J) for community-dwelling cancer patients. Am J Hosp Palliat Care. 2009;26(5):334–40. 10.1177/1049909109338480 19648575

[38] Malhotra R, Chan A, Malhotra C, Østbye T (2012). Validity and reliability of the Caregiver Reaction Assessment scale among primary informal caregivers for older persons in Singapore. Aging Ment Health.

[39] Nunnally JC, Bernstein IH (1994). Psychometric theory.

[40] Kroenke K, Spitzer RL, Williams JBW (2001). The PHQ-9: validity of a brief depression severity measure. J Gen Intern Med.

[41] American Psychiatric Association. Diagnostic and statistical manual of mental disorders. 4th ed. Washington DC; 1994

[42] Kroenke K, Spitzer RL, Williams JB, Lowe B (2010). The patient health questionnaire somatic, anxiety, and depressive symptom scales: a systematic review. Gen Hosp Psychiatry.

[43] Perkins EA (2010). The compound caregiver: a case study of multiple caregiving roles. Clin Gerontol.

[44] Yun-Hee J, Brodaty H, Chesterson J (2005). Respite care for caregivers and people with severe mental illness: literature review. J Adv Nurs.

[45] Cheung SF, Cheung FM, Howard R, Lim Y-H (2006). Personality across the ethnic divide in Singapore: are “Chinese Traits” uniquely Chinese?. Personal Individ Differ.

[46] Phua VC, Loh J (2008). Filial piety and intergenerational co-residence: the case of Chinese Singaporeans. Asian J Soc Sci.

[47] Cicirelli VG (1993). Attachment and obligation as daughters’ motives for caregiving behavior and subsequent effect on subjective burden. Psychol Aging.

[48] Yueh-Feng Lu Y, Austrom MG (2005). Distress responses and self-care behaviors in dementia family caregivers with high and low depressed mood. J Am Psychiatr Nurses Assoc.

[49] Pearlin LI, Mullan JT, Semple SJ, Skaff MM (1990). Caregiving and the stress process: an overview of concepts and their measures. Gerontologist.

[50] de Wit LM, Fokkema M, van Straten A, Lamers F, Cuijpers P, Penninx BW (2010). Depressive and anxiety disorders and the association with obesity, physical, and social activities. Depress Anxiety.

[51] Teychenne M, Ball K, Salmon J (2008). Physical activity and likelihood of depression in adults: a review. Prev Med.

[52] Roh HW, Hong CH, Lee Y, Oh BH, Lee KS, Chang KJ, Kang DR, Kim J, Lee S, Back JH et al. Participation in physical, social, and religious activity and risk of depression in the elderly: a community-based three-year longitudinal study in Korea. PLoS One. 2015;10(7), e0132838. 10.1371/journal.pone.0132838 PMC4501692 26172441

[53] Braun M, Mikulincer M, Rydall A, Walsh A, Rodin G (2007). Hidden morbidity in cancer: spouse caregivers. J Clin Oncol Off J Am Soc Clin Oncol.

[54] Costa-Requena G, Cristófol R, Cañete J (2012). Caregivers’ morbidity in palliative care unit: predicting by gender, age, burden and self-esteem. Support Care Cancer.

[55] Salin S, Kaunonen M, Åstedt-Kurki P (2009). Informal carers of older family members: how they manage and what support they receive from respite care. J Clin Nurs.

[56] Mensie LC, Steffen AM (2010). Depressive symptoms and use of home-based respite time in family caregivers. Home Health Care Serv Q.

[57] Moullin S (2007). Care in a New welfare society: unpaid care, welfare and employment.

[58] Yeh P-M, Chang Y (2012). Family carer reactions and their related factors among Taiwanese with hospitalized relatives. J Adv Nurs.

[59] Lehan T, Arango-Lasprilla JC, Macias MA, Aguayo A, Villasenor T (2012). Distress associated with patients’ symptoms and depression in a sample of Mexican caregivers of individuals with MS. Rehabil Psychol.

[60] Covinsky KE, Newcomer R, Fox P, Wood J, Sands L, Dane K, Yaffe K. Patient and caregiver characteristics associated with depression in caregivers of patients with dementia. J Gen Intern Med. 2003;18(12):1006–14. 10.1111/j.1525-1497.2003.30103.x PMC1494966 14687259

[61] Tessler R, Gamache G (1994). Continuity of care, residence, and family burden in Ohio. Milbank Q.

[62] Crowe A, Brinkley J (2015). Distress in caregivers of a family member with serious mental illness. Fam J.

